# Is fantasy baseball score a viable outcome measure for professional baseball pitchers after undergoing Tommy John surgery?

**DOI:** 10.1016/j.jseint.2022.09.018

**Published:** 2022-10-29

**Authors:** Trevor G. Simcox, Vivek Singh, Jack Tesoriero, Gennaro DelliCarpini, Mandeep S. Virk, Ioannis Zouzias, Mark G. Grossman

**Affiliations:** a Department of Orthopedic Surgery, NYU Langone Health – Long Island School of Medicine, Mineola, NY, USA; b Department of Orthopedic Surgery, NYU Langone Health, New York, NY, USA; c New York Medical College, Valhalla, NY, USA

**Keywords:** Ulnar collateral ligament, UCL reconstruction, Major league baseball, MLB pitchers, Fantasy baseball score, Baseball injury, Elbow surgery, Orthopedic outcomes

## Abstract

**Hypothesis and Background:**

Although on-field performance metrics are useful in measuring overall success of ulnar collateral ligament (UCL) reconstruction (UCLR) in professional baseball pitchers, they may not comprehensively quantify athletic performance after returning to playing in the league. To utilize fantasy baseball score (FBS) as a novel and objective outcome to assess the quality of return to play in major league baseball (MLB) pitchers who went back to professional pitching after UCLR.

**Methods:**

This is a retrospective observational cohort study of 216 established MLB pitchers who underwent UCLR while in the MLB between the years 1974 and 2018. Pitchers who either started in at least 45 games or pitched 90 relief games in the 3 years leading up to injury were included. FBS was calculated using 3 different scoring methods: ESPN (Entertainment and Sports Programming Network) (FBS-ESPN), Yahoo (FBS-Yahoo), and CBS (Columbia Broadcasting System) (FBS-CBS). Return to play, games played, innings pitched, earned runs, strikeouts, walks, hits allowed, hit batsman, and quality starts were also collected. Performance records were compiled for 3 years prior to and after the return to MLB. Players' pre-injury performance was used as a historic control group. Multivariate linear regression analysis was used to detect trends between seasons, controlling for year of surgery, and number of games.

**Results:**

The mean age of the cohort at the time of surgery was 30.0 ± 3.5 years. One hundred seventy-nine of 216 players (83%) returned to MLB play, taking an average of 16.6 ± 5.8 months. One hundred thirty-six of 179 (76%) remained in the league for 3 seasons. After adjusting for year of surgery, pitchers earned significantly fewer points for FBS-CBS (616.45 ± 332.42 vs. 389.12 ± 341.06; *P* < .001), FBS-Yahoo (801.90 ± 416.88 vs. 492.57 ± 428.40; *P* < .001), and FBS-ESPN (552.76 ± 275.77 vs. 344.19 ± 300.45; *P* < .001) after their injury. Also pitchers played in fewer games, pitched fewer innings, and had a decline in all measured on-field performance statistics.

**Conclusion:**

FBS may represent a useful outcome measure to objectively assess the quality of return to play in a professional baseball pitcher who returned to play in MLB after UCLR.

Elbow ulnar collateral ligament (UCL) reconstruction (UCLR) has become increasingly utilized in the treatment of recreational and major league baseball (MLB) pitchers after UCL rupture.^23^ Primary UCLR is generally successful in terms of return to play, patient-reported outcomes (PROs), and performance statistics for professional pitchers. The surgical technique of UCLR has evolved since its inception in 1974^1^^,^^15^^,^^24^^,^^26^ and overhead athletes have achieved excellent results, reported via the Conway score,^6^ 83% of the time with an overall complication rate of 10%.^3^^,^^8^^,^^18^^,^^22^^,^^24^^,^^27^ Despite players having generally positive outcomes after UCLR, there still exist significant barriers for professional players to return to a pre-injury level of play.^5^

Surgical success has been reported using several injury-specific PROs. One such score is the Conway score which grades the athlete as poor, fair, good, and excellent depending on their ability to return to prior level of play.^6^ Another is the Kerlan-Jobe Orthopaedic Clinic (KJOC) score, consisting of a subjective questionnaire that grades an overhead athlete’s functional performance.^9^ Although these outcome measures are useful in measuring overall success, they may lack the ability to comprehensively quantify athletic performance amongst pitchers who have returned to the MLB, the highest level of play. Studies have also reported outcomes in terms of on-field performance statistics such as earned run average, innings pitched, etc.^21^ Individual on-field performance metrics may act as a surrogate for overall performance, but no study to date has created an outcome measure that integrates multiple on-field performance metrics into one score.

Fantasy Baseball Score (FBS) has been shown to be reproducible and predictive of future performance on a season-to-season basis,^13^^,^^14^^,^^19^ we propose that FBS may be used as an objective outcome measure in high-performing professional baseball athletes. Fantasy points represent an objective and comprehensive measure of on-field performance as compared to individual performance statistics, which may provide an incomplete picture of overall performance. The purpose of this study is to demonstrate that FBS may be used to measure quality of return to play, from which elbow surgeons may use to counsel MLB pitchers regarding their expected performance outcomes after UCLR.

## Materials and methods

### Generation of study cohort

This is a retrospective observational cohort study of established MLB pitchers who underwent UCLR while in the MLB. In order to generate our study cohort, players were identified as having undergone UCLR using the following baseball databases: “Baseball Reference”, “Fangraphs”, “Baseball Cube”, MLB.com, and rostersource.com. Verification of UCLR was performed by one of the authors by searching for a corresponding publicly available press release. Due to the detrimental nature of UCL tear on professional performance, the MLB pitchers who have undergone UCLR are well documented in the public record (team press releases, newsprint, recorded statements by player, etc). All data is publicly available, and this study is exempt from institutional review board approval.

Between the years 1974 and 2018, 426 players were identified as having undergone primary UCLR during their MLB career. Pitchers who did not start a minimum of 45 games or relief pitch in 90 games in 3 seasons prior to injury were excluded. These exclusion criteria were necessary to establish reliable baseline MLB performance prior to injury, which would be used as paired historic control for postoperative performance. Prior sabermetrics research (i.e., empirical analyses of baseball statistics) has found that FBS for pitchers is predictive of future performance on a season-to-season basis^13^^,^^19^ but there is high variance in scores with shorter playing time. The pitching position, compared with other positions, is subjected to high variability in score on a week-to-week basis. Any given pitcher may have a lapse in their gameplay and get pulled early from the game having accrued high numbers of negative points in a short period of time. Because of this phenomenon specific to MLB pitchers, the cutoff for minimum number of games played needed to be sufficiently high to use FBS as a mathematically reliable measure of baseline performance.^14^ Two hundred ten pitchers failed to meet the inclusion and exclusion criteria, leaving a total of 216 players for analysis.

### Data collection

The primary outcome studied was FBS, calculated using the following 3 different scoring methods from the most popular fantasy baseball websites: ESPN (Entertainment and Sports Programming Network) (FBS-ESPN), Yahoo (FBS-Yahoo), and CBS (Columbia Broadcasting System) (FBS-CBS). Table I lists pitching performance statistics used to tabulate score as well as the relative weights of each performance statistic. Scores were calculated by the authors using raw data from baseball database records. Using this methodology fantasy scores were generated for all pitchers, even those who played prior to the advent of fantasy baseball but who had sufficient records in the MLB databases.

**Table I:** On-field performance metrics included in each fantasy baseball scoring system and the points awarded for each on-field metric.

Fantasy scoring system	Earned run	Strike out	Walk	Hit allowed	Inning pitched	Hit batsman	Quality start
Entertainment and Sports Programming Network	−2	1	−1	−1	3	Not Included	Not Included
Yahoo	−3	3	−1.3	−1.3	3	−1.3	Not Included
Columbia Broadcasting System	−1	0.5	−1	−1	3	−1	3

Secondary outcome measures were return to MLB play, time of return to MLB play, games played, innings pitched, earned runs, strikeouts, walks, hits allowed, hit batsman, and quality starts. Return to MLB play was defined as playing in at least 1 MLB game after surgery. Quality starts are awarded to a player for pitching at least six innings and allowing less than 3 runs. Baseball-Reference.com was used to compile player demographics and performance statistics. The number of quality-starts was obtained from MBL.com. Performance records were compiled for 3 years prior to and 3 years after return to MLB. Nomenclature of seasons is relative to season of injury and return to MLB. For example, “pre-injury season 3” is in reference to 3 seasons prior to season of injury and “post-surgery season 3” refers to the third season after return to MLB. Fantasy score and performance statistics were recorded as per the season totals.

### Statistical analysis

Descriptive data are represented as means ± standard deviation (SD). Statistical differences in numeric and continuous variables were detected using paired 2-sided t-tests and analysis of variance (ANOVA), whereas a player’s pre-injury performance was used as a historic control for post-surgery performance. Multivariate linear regressions were used to detect trends between the 6 baseball seasons (3 pre-injury and 3 post-surgery). A multivariate model was used to adjust for the year of surgery and number of games played due to the evolution in surgical technique and to account for the differences in the number of games a pitcher played before and after undergoing UCLR. Subgroup analysis of FBS, by decade, was performed by categorizing pitchers based on date of surgery into 1 of 3 subgroups as follows: before 2000, between 2001 and 2010, and 2011 to present. This analysis was performed to assess the general effect of evolving surgical technique and rehabilitation protocols on outcomes. A *P* value of less than .05 was considered to be significant. All statistical analyses were performed using SPSS, version 25 (IBM Corporation, Armonk, NY, USA).

## Results

### Demographics and return to play

There were 426 pitchers who underwent UCLR while playing in the MLB and 216 met inclusion criteria for the study. At time of surgery, players were a mean age of 30.0 ± 3.5 years. Return to play analysis revealed that 179 of 216 players (83%) returned to MLB play, taking an average of 16.6 ± 5.8 months to return. Of the 179 players who returned to play in the MLB, only 136 (63%) remained in the league for 3 seasons after the season of injury.

### Fantasy Baseball Score before and after injury

There was a significant decline in the number of fantasy points accrued when comparing the 3 seasons before injury to the 3 seasons after MLB return. After adjusting for year of surgery, pitchers earned significantly fewer points for FBS-CBS (616.45 ± 332.42 vs. 389.12 ± 341.06; *P* < .001), FBS-Yahoo (801.90 ± 416.88 vs. 492.57 ± 428.40; *P* < .001), and FBS-ESPN (552.76 ± 275.77 vs. 344.19 ± 300.45; *P* < .001) after their injury. Regarding individual on-field performance statistics, after surgery pitchers played in fewer games (120.68 ± 57.52 vs 72.59 ± 30.77, *P* < .001), pitched fewer innings (338.06 ± 170.64 vs 223.56 ± 165.01, *P* < .001), and had a decline in all measured on-field performance statistics including earned runs (144.02 ± 84.54 vs. 102.13 ± 73.02; *P* < .001), hits allowed (327.41 ± 181.83 vs. 218.83 ± 166.42; *P* < .001), walks (116.32 ± 60.63 vs. 78.52 ± 51.32; *P* < .001), hit batsman (11.12 ± 7.76 vs. 8.09 ± 7.15; *P* < .001), strikeouts (270.36 ± 128.38 vs. 175.12 ± 126.20; *P* < .001), and quality starts (22.00 ± 22.46 vs. 12.82 ± 18.52; *P* < .001). All metrics remained significantly different after controlling for both year and number of games played. Table II details pre-injury and post-surgery raw on-field performance metrics (Table IIA), as well as FBS totals and component scores for FBS-ESPN (Table IIB), FBS-Yahoo (Table IIC), and FBS-CBS (Table IID)

**Table II:** Comparison of on-field performance and fantasy points accrued over 3 seasons prior to season of injury and First 3 major league baseball Seasons after ulnar collateral ligament (n = 136).

	A: Raw on-field performance		
	Before injury	Post-surgery	*P* value
Total games played	120.68 ± 57.52	72.59 ± 30.77	<.001
Total innings pitched	338.06 ± 170.64	223.56 ± 165.01	<.001
Total earned runs	144.02 ± 84.54	102.13 ± 73.02	<.001
Total hits allowed	327.41 ± 181.83	218.83 ± 166.42	<.001
Total walks	116.32 ± 60.63	78.52 ± 51.32	<.001
Total hits batsman	11.12 ± 7.76	8.09 ± 7.15	.001
Total strikeouts	270.36 ± 128.38	175.12 ± 126.20	<.001
Total quality starts	22.00 ± 22.46	12.82 ± 18.52	<.001
Total fantasy points			

	B: Entertainment and Sports Programming Network fantasy points comparison		
	Before injury	Post-surgery	*P* value
Total games played			
Total innings pitched	1014.17 ± 511.91	670.68 ± 495.03	<.001
Total earned runs	−288.03 ± 169.08	−204.26 ± 146.05	<.001
Total hits allowed	−327.41 ± 181.83	−218.83 ± 166.42	<.001
Total walks	−116.32 ± 60.63	−78.52 ± 51.32	<.001
Total hits batsman			
Total strikeouts	270.36 ± 128.38	−175.12 ± 126.20	<.001
Total quality starts			
Total fantasy points	552.76 ± 275.77	344.19 ± 300.45	<.001

	C: Yahoo fantasy points comparison		
	Before injury	Post-surgery	*P* value
Total games played			
Total innings pitched	1014.17 ± 511.91	670.68 ± 495.03	<.001
Total earned runs	−432.05 ± 253.62	−306.39 ± 219.06	.019
Total hits allowed	−425.63 ± 236.38	−284.48 ± 216.35	<.001
Total walks	−151.22 ± 78.83	−102.08 ± 66.72	<.001
Total hits batsman	−14.46 ± 10.09	−10.51 ± 9.29	.001
Total strikeouts	811.09 ± 385.15	525.35 ± 378.59	<.001
Total quality starts			
Total fantasy points	801.90 ± 416.88	492.57 ± 428.40	<.001

	D: Columbia Broadcasting System fantasy points comparison		
	Before injury	Post-surgery	*P* value
Total games played			
Total innings pitched	1014.17 ± 511.91	670.68 ± 495.03	<.001
Total earned runs	−144.02 ± 84.54	−102.13 ± 73.02	<.001
Total hits allowed	−327.41 ± 181.83	−218.83 ± 166.42	<.001
Total walks	−116.32 ± 60.63	−78.52 ± 51.32	<.001
Total hits batsman	−11.12 ± 7.76	−8.09 ± 7.15	.001
Total strikeouts	135.18 ± 64.19	87.56 ± 63.10	<.001
Total quality starts	65.98 ± 67.37	38.45 ± 55.57	<.001
Total fantasy points	616.45 ± 332.42	389.12 ± 341.06	<.001

### By-season comparison of major league baseball performance

**Figure 1:**
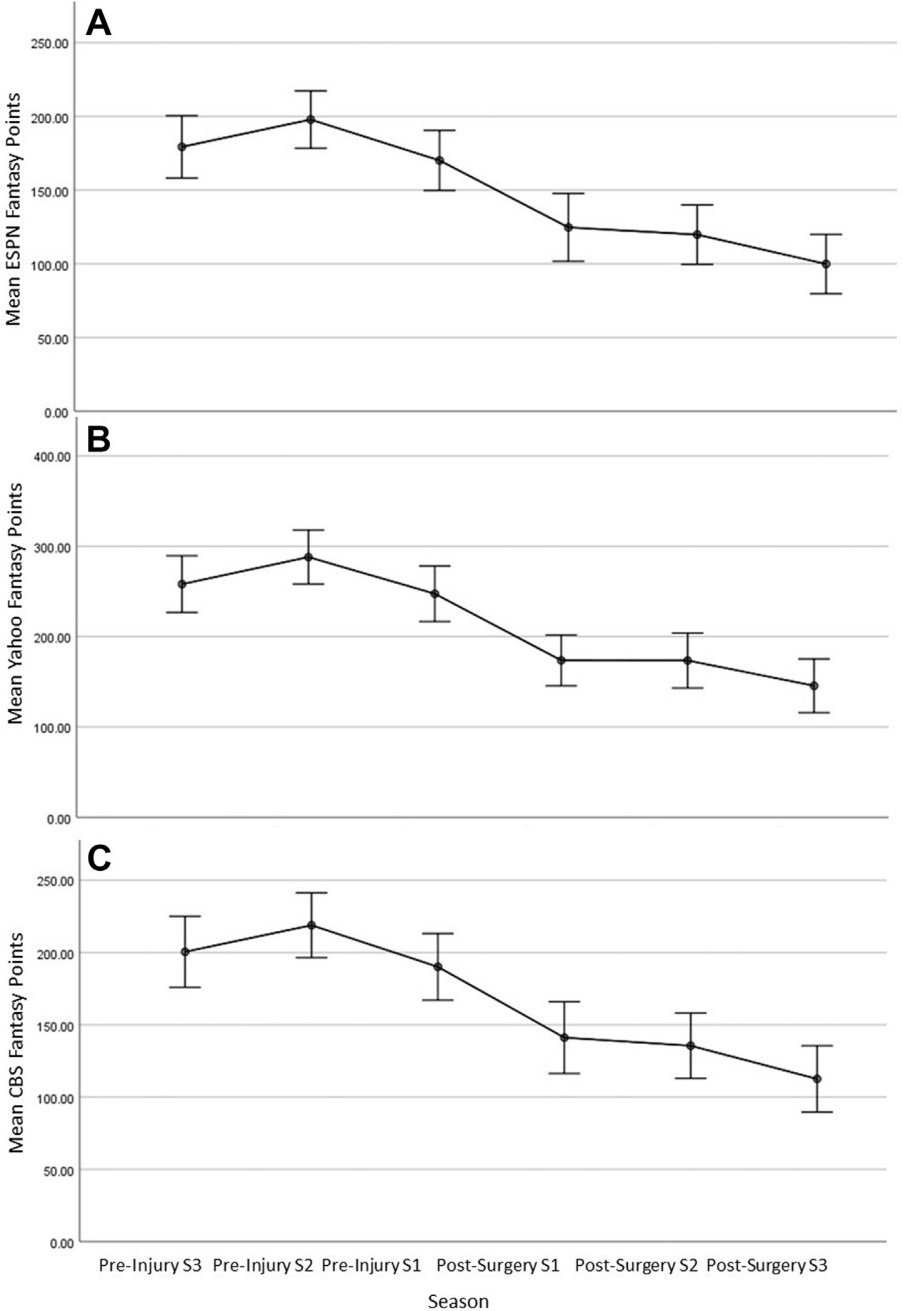
Line graph of mean total fantasy baseball score (FBS) per season. Error bars represent 95% confidence interval. Pre-injury season 1 (S1) denotes the season immediately prior to the season of injury and post-surgery S1 denotes the first season in which a pitcher returned to play in the major league baseball. Multivariate linear regression analysis depicts down-trending mean FBS-Entertainment and Sports Programming Network (**A**), FBS-Yahoo (**B**), and FBS-Columbia Broadcasting System (**C**) between pre-injury season 2 through post-surgery season 3. These associations were maintained despite controlling for number of games played per season and decade of surgery.

Primary and secondary outcomes were then compared by season using multivariate linear regression analysis to control for both decade of surgery and number of games played (Table III, Fig. 1, A-*C*). This analysis revealed that after undergoing UCLR, pitchers suffered a significant decline between pre-injury season 1 and post-surgery season 1 in games played (*P* < .001), innings pitched (*P* < .001), earned runs (*P* < .001), hits allowed (*P* < .001), walks (*P* < .001), hit batsman (*P* < .001), strikeouts (*P* < .001), quality starts (*P* = .001), FBS-CBS (*P* < .001), FBS-ESPN (*P* < .001), and FBS-Yahoo (*P* < .001). The decline in FBS between pre-injury and post-surgery was maintained for post-surgery seasons 2 and 3 (Fig. 1). For each of the FBS scoring methods a decline in performance was noted between pre-injury season 2 and season 1, leading up to the season of injury.

**Table III:** Comparison of pre-injury to post-surgery on-field performance and fantasy points by season.

	Pre-injury S3	Pre-injury S2	Pre-injury S1	Post-surgery S1	Post-surgery S2	Post-surgery S3	*P* value
Games played	35.5 ± 27.4	44.0 ± 25.0	41.2 ± 21.9	34.3 ± 16.7	37.4 ± 21.1	34.5 ± 20.1	.001
Innings pitched	115.1 ± 64.8	119.2 ± 66.5	107.8 ± 70.8	82.0 ± 63.7	84.9 ± 63.6	86.4 ± 68.5	<.001
Earned runs	48.0 ± 30.9	50.0 ± 33.4	47.3 ± 36.3	36.4 ± 27.5	37.1 ± 29.3	28.7 ± 31.7	<.001
Hits allowed	110.2 ± 66.2	114.8 ± 69.3	106.5 ± 75.8	78.1 ± 58.9	83.8 ± 62.9	86.5 ± 71.7	<.001
Walks	38.6 ± 23.0	41.2 ± 24.1	38.9 ± 26.8	28.7 ± 18.1	31.4 ± 22.2	28.5 ± 21.1	<.001
Hit batsman	3.2 ± 2.7	4.1 ± 3.4	3.9 ± 3.8	2.9 ± 2.5	3.4 ± 3.6	2.8 ± 2.9	.004
Strikeout	90.3 ± 48.7	96.2 ± 52.4	86.7 ± 55.1	60.3 ± 40.0	63.6 ± 53.5	51.2 ± 55.6	<.001
Quality start	7.7 ± 8.6	7.7 ± 8.5	6.6 ± 7.9	4.4 ± 6.4	4.9 ± 7.4	5.3 ± 7.8	.001
Columbia Broadcasting System fantasy points	200.5 ± 138.3	218.9 ± 132.3	190.1 ± 136.0	141.1 ± 146.5	135.5 ± 133.2	112.5 ± 135.3	<.001
Entertainment and Sports Programming Network fantasy points	179.3 ± 119.0	197.8 ± 114.6	170.0 ± 120.2	124.7 ± 135.7	119.7 ± 118.8	99.8 ± 118.5	<.001
Yahoo fantasy points	258.0 ± 176.4	288.0 ± 176.1	247.4 ± 181.3	173.6 ± 165.6	173.5 ± 179.2	145.5 ± 174.7	<.001

*S1*, Season 1; *S2*, Season 2; *S3*, Season 3

### Sub-group analysis by decade

A sub-group analysis, comparing fantasy performance by decade, was performed to determine if decade of surgery has a significant impact on postsurgical FBS. The subgroups pre-2000, 2001 to 2010, and 2011 to present were compared to reveal that the trend towards lower post-surgery FBS was maintained between the subgroups (Table IV).

**Table IV:** Subgroup analysis by decade of fantasy points production pre-injury and post-surgery.

	Pre-injury S3	Pre-injury S2	Pre-injury S1	Post-surgery S1	Post-surgery S2	Post-surgery S3	*P*-value
Surgery prior to 2000 (n = 29)							
CBS fantasy points	235.71 ± 138.32	233.02 ± 124.77	164.19 ± 127.45	122.76 ± 84.47	126.16 ± 131.46	117.79 ± 122.93	<.001
ESPN fantasy points	200.62 ± 116.68	196.79 ± 101.68	134.35 ± 103.73	105.59 ± 67.00	106.86 ± 115.46	103.00 ± 106.40	<.001
Yahoo fantasy points	261.53 ± 159.41	257.07 ± 140.35	169.29 ± 132.29	135.17 ± 86.74	142.40 ± 164.29	146.14 ± 154.57	<.001
Surgery between 2001-2010 (n = 46)							
CBS fantasy points	198.20 ± 139.55	208.32 ± 140.84	198.09 ± 145.16	164.44 ± 196.19	143.15 ± 136.68	100.42 ± 133.03	.002
ESPN fantasy points	177.11 ± 121.15	188.69 ± 119.96	176.61 ± 129.96	142.86 ± 187.38	124.98 ± 120.80	87.56 ± 113.00	.001
Yahoo fantasy points	254.47 ± 182.64	270.32 ± 178.86	253.81 ± 198.76	188.20 ± 208.81	175.45 ± 180.04	122.16 ± 157.90	<.001
Surgery after 2011 (n = 52)							
CBS fantasy points	184.86 ± 136.76	221.34 ± 129.22	196.15 ± 132.08	128.72 ± 112.80	133.14 ± 132.90	121.22 ± 144.75	<.001
ESPN fantasy points	170.54 ± 119.24	206.94 ± 117.03	182.65 ± 117.07	117.49 ± 100.02	121.56 ± 120.38	109.69 ± 130.19	<.001
Yahoo fantasy points	259.42 ± 182.05	320.94 ± 187.46	282.41 ± 176.59	180.08 ± 149.81	187.94 ± 186.86	167.25 ± 198.46	<.001

*CBS*, Columbia Broadcasting System; *ESPN*, Entertainment and Sports Programming Network; *S1*, Season 1; *S2*, Season 2; *S3*, Season 3

## Discussion

Our study of established MLB pitchers who have undergone UCLR found the overall rate of return to MLB to be 83%, but only 63% of the cohort remained in the MLB after 3 seasons. Of the pitchers who continued playing for at least 3 seasons, there was a significant decline in performance both in their first season returning and in the subsequent 2 seasons thereafter. Our study also showed a decline in fantasy performance leading up to their injury as follows: a decline from pre-injury season 2 to season 1.

Although return to same level of play is an essential outcome measure, it is equally important to objectively assess quality of return to play. To date, there is a paucity of comprehensive tools used to assess on-field athletic performance in MLB pitchers. PROs and pitching velocity have traditionally represented a useful way to measure surgical success, but KJOC or Conway scores may not directly correlate with on-field performance as they may lack the granularity to distinguish between high, mediocre, and poor performance levels once return to MLB has been achieved. Furthermore, the measurement of singular on-field performance metrices may fail to illustrate a complete picture of an individual’s performance. We propose that FBS may be used as a measure of a professional athlete’s overall performance and is a helpful adjunct to already established outcome measures that are currently used in this patient population. Fantasy baseball was initially popularized in the 1980s and although there are various methods for tabulating points, FBS is well established by fans, sabermetric researchers,^13^^,^^14^^,^^19^ and in the sports betting industry as an objective measure of on-field performance.^2^^,^^17^^,^^25^ The value of this study is in demonstrating that FBS may be used as a valuable surrogate measure for athletic performance before and after injury.

Prior studies have demonstrated that MLB pitchers return to sport at a rate of 80%-95% ^7^^,^^10^, ^11^, ^12^^,^^16^^,^^20^^,^^21^ on an average of 11.6-18.5 months after surgery .^4^^,^^12^^,^^16^^,^^18^^,^^20^^,^^22^ For our cohort, return to MLB level of play was 83% on an average of 16.6 months after surgery. It is also worth noting that 44 of the 216 (20%) of pitchers were able to return for at least 1 game but subsequently retired within 3 seasons.

The current literature illustrates mixed outcomes regarding on-field performance after UCLR, with some studies reporting similar or improved performance after UCLR^12^^,^^21^ while others report a postoperative decline.^16^^,^^20^ Our study is the most consistent with findings of Makhni et al, who found a sustained postoperative decline in performance. Makhni et al noted that of the 80% of pitchers who returned to MLB play, 57% returned to the disabled list and there was diminished performance in the 2 seasons following return to play.^16^ This contradicts the findings of Marshall et al who found that pitching performance measures such as pitching velocity, innings pitched, earned runs average, and walks and hits per inning pitched returned to preoperative levels by the second year postoperatively.^21^ Interestingly, our study showed a decline in fantasy performance leading up to their injury (a decline from pre-injury season 2 to season 1). This phenomenon was observed in prior studies by Erickson and Keller et al.^11^^,^^16^ A key difference to note is that our exclusion criteria eliminated a large number of pitchers who failed to establish a sufficient MLB track record prior to injury. Consequently, our findings as they relate to UCLR outcomes may have limited generalizability to all pitchers who are indicated for the procedure.

In our study population, there was a postoperative decline in games played, innings pitched, and all on-field performance metrics including earned runs, hits allowed, walks, hit batsman, strikeouts, and quality starts. There was also a subsequent rebound in performance between post-injury season 1 and season 3 with regard to innings pitched, earned runs, hits allowed, and quality starts. Keller et al identified a similar trend where MLB pitchers exhibited an initial performance decline postoperatively, but their performance rebounded in the subsequent 2 seasons. Although, in Keller et al’s cohort, most pitchers failed to achieve their pre-injury performance level.^16^ For our cohort, FBS declined despite there being a discordance in improvement for different performance statistics, suggesting that changes in FBS were attributable to both pitching fewer innings per game and reduced overall performance while on-field. This may indicate that FBS appears to act as a barometer for a player’s overall performance throughout a season, capturing both a decline in innings pitched and quality of play.

In order to account for evolution in operative technique over the years of the study, we performed a by-decade subgroup analysis to examine if pitchers fared better in recent decades as compared to those prior. In general, the overarching trend in which pitchers experience a preoperative decline from pre-injury season 2 to season 1, followed by a substantial decline for post-surgery season 1 is maintained. One notable divergence from this trend was noted for the 2011 to present subgroup, where there was an increase in FBS from post-surgery season 1 to 2, followed by a significant decline in season 3—a similar trend was seen in some studies conducted during the same time period.^16^^,^^21^

Our study has several notable limitations that must be recognized. As this is a retrospective observational study of publicly available data, important surgical details such as experience of the operating surgeon, technique utilized, concomitant procedures, and rehabilitation protocol were not available. To adjust for this, all analyses have been adjusted for year of surgery in order to offset this effect. We also assumed that surgeons treating MLB pitchers utilize fixation techniques and rehabilitation protocols that are consistent with the standard of care. Conway and KJOC scores or pitching velocity are not publicly available, and we are unable to correlate FBS to these previously established outcomes. It is possible that the injury database did not reveal every pitcher who underwent UCLR. Although this limitation is mitigated by the fact that UCLR is a major operation, it requires years to return to play, resulting in the public knowledge of nearly all UCLR procedures for MLB players. Our exclusion criteria eliminated 210 MLB pitchers who had not played in a sufficient number of games to reliably calculate FBS. Furthermore, most MLB pitchers who have undergone UCLR did so prior to professional play. The mean age of our study population was 30 years, and the mean age of MLB debut is roughly 25 years, suggesting that our cohort is comprised of established pitchers. Pitchers who are still in the league at the age 30 have an MLB retention rate of 80.8% at 4-years. This is likely due to the fact that established pitchers may have a larger repertoire of pitches and have an improved capacity to adjust their game after UCLR as they age, and experience diminished fastball speed. Whereas less experienced pitchers who undergo UCLR may be more susceptible to worse play in the context of diminishing fastball speed as they age. This must be considered because our outcome data may not necessarily represent all of the MLB pitchers who underwent this operation. Rather, our study design was aimed to demonstrate that FBS may be a useful measure of performance rather than to reiterate outcomes after UCLR surgery.

## Conclusions

FBS may represent a useful outcome measure in the assessment of professional baseball pitchers’ quality of return to play, as it combines several on-field performance statistics into a singular objective score. This may augment the criterion that surgeons currently use to measure surgical success for UCLR. Further research may focus on FBS as a predictor of return to play and validation of FBS as an outcome measure for professional baseball players.

## Disclaimers

Funding: The authors and institutions received no financial support for the research, authorship, and/or publication of this article.

Conflicts of interest: Dr. Virk is a paid consultant for Exactech, Inc. regarding an unrelated topic to this study. The other authors, their immediate families, and any research foundations with which they are affiliated have not received any financial payments or other benefits from any commercial entity related to the subject of this article.
